# The efficacy and safety of upadacitinib in atopic dermatitis: A systematic review and meta-analysis

**DOI:** 10.1097/MD.0000000000045239

**Published:** 2025-10-17

**Authors:** Amr Molla, Mohammed Alahmadi, Sara Alghamdi, Abdulsalam Humedi, Maryam Alfaraj, Basel Bakhamees, Samia Khalil, Salma Alhussaini

**Affiliations:** a Department of Medicine, College of Medicine, Taibah University, Medina, Saudi Arabia; b College of Medicine, Taibah University, Medina, Saudi Arabia; c College of Medicine, Al-Baha University, Al-Baha, Saudi Arabia; d College of Medicine, Jazan University, Jazan, Saudi Arabia; e Collage of Medicine, Imam Abdulrhman Bin Faisal University, Dammam, Saudi Arabia; f College of Medicine, King Abdulaziz University, Jeddah, Saudi Arabia; g College of Medicine, Ibn Sina National College, Jeddah, Saudi Arabia.

**Keywords:** atopic dermatitis, efficacy, JAK inhibitor, meta-analysis, safety, systematic review, upadacitinib

## Abstract

**Background:**

Atopic dermatitis (AD) is a chronic inflammatory skin condition with a significant disease burden. Systemic therapies, including Janus kinase inhibitors, offer targeted treatment options for moderate-to-severe AD. Upadacitinib (UPA) is a selective Janus kinase 1 inhibitor which has demonstrated promising efficacy in clinical trials. This systematic review and meta-analysis evaluate the efficacy and safety of UPA in AD treatment.

**Methods:**

A comprehensive systematic search was conducted across the following databases: PubMed, Web of Science, and Google Scholar, following PRISMA guidelines. Randomized controlled trials evaluating UPA for moderate to severe AD were included. Efficacy outcomes included Eczema Area and Severity Index (EASI) improvement (EASI-75, EASI-90), pruritus reduction, and overall treatment response. Safety outcomes assessed the adverse events and tolerability. A meta-analysis was performed using a random-effects model to estimate pooled RRs and confidence intervals (CIs).

**Results:**

Five randomized controlled trials met the inclusion criteria with a total of 2208 participants (1153 in the treatment group and 1055 in the control group). UPA significantly improved EASI-75 response rates compared to placebo or dupilumab (pooled RR = 1.77, 95% CI: 1.34–2.33; *P* < .0001), and showed a superior EASI-90 response (pooled RR = 3.83, 95% CI: 2.17–6.78; *P* < .0001). Pruritus numeric rating scale scores improved significantly (RR = 1.98, 95% CI: 1.42–2.76; *P* < .0001). Subgroup analysis revealed that both UPA 15 mg and 30 mg were superior to placebo or dupilumab, with EASI-75 subgroup RRs of 2.64 (95% CI: 0.84–8.34) and 1.95 (95% CI: 1.09–3.51), respectively. Similarly, EASI-90 responses were markedly higher with 15 mg (RR = 8.70, 95% CI: 3.60–21.02) and 30 mg (RR = 6.01, 95% CI: 0.90–40.03), with greater effect observed at the higher dose.

**Conclusion:**

UPA is an effective systemic treatment for moderate-to-severe AD, demonstrating rapid and sustained symptomatic improvement with an acceptable safety profile. While its efficacy exceeds the current biological therapies in some aspects, long-term safety monitoring and comparative trials are needed to optimize treatment strategies.

## 1. Introduction

Atopic dermatitis (AD) is a chronic inflammatory skin disorder that primarily affects individuals with a genetic predisposition and is often exacerbated by environmental triggers. The disease has a multifactorial pathogenesis involving immune dysregulation, genetic mutations, skin barrier dysfunction, and environmental factors. AD is characterized by thickened skin, persistent pruritus that worsens at night, and intensely itchy papules, which may exude clear fluid when scratched.^[1]^ The prevalence of AD is estimated at approximately 20% in children and 1% to 3% in adults, with a higher incidence in females. The condition follows a bimodal distribution, with peaks occurring in early childhood and middle adulthood.^[2]^ Global disease burden studies from 1990 to 2017 ranked AD as the leading cause of disability-adjusted life years among skin diseases and the 15th most burdensome condition worldwide. Severity is commonly assessed using tools such as the Scoring Atopic Dermatitis and the Eczema Area and Severity Index (EASI).^[3,4]^

The standard treatment for mild AD includes emollients and topical corticosteroids, which are often sufficient for symptom control. However, moderate-to-severe AD frequently necessitates systemic immunosuppressants, which, despite their effectiveness, pose risks such as myelosuppression and hepatic or renal impairment with long-term use (1). The advent of targeted biologic therapies has provided a safer alternative, with monoclonal antibodies offering improved efficacy in managing uncontrolled AD. Dupilumab, an interleukin (IL)-4 receptor antagonist, was the first biologic approved for AD and has demonstrated both efficacy and a favorable safety profile.^[4,5]^ Nevertheless, a subset of patients fails to achieve adequate disease control with dupilumab, necessitating alternative therapeutic approaches.^[6]^

Recent advancements have introduced Janus kinase (JAK) inhibitors as a novel class of targeted therapies for AD. The Janus kinase-signal transducer and activator of transcription signaling pathway plays a critical role in mediating Th2-driven inflammation in AD by regulating cytokines such as IL-4, IL-13, and IL-31. Upadacitinib (UPA), an oral selective JAK1 inhibitor, exhibits higher specificity for JAK1 than JAK2, JAK3, or tyrosine kinase 2.^[7]^ Initially approved in 2019 for moderate-to-severe rheumatoid arthritis in patients with inadequate responses to methotrexate, UPA received Food and Drug Administration approval in 2022 for the treatment of moderate-to-severe AD in adolescents and adults. Clinical trials have demonstrated significant improvements in skin clearance and pruritus reduction with UPA.^[8–10]^

Given the growing body of research supporting UPA’s efficacy, this study aims to provide a comprehensive, up-to-date systematic review (SR) and meta-analysis evaluating its effectiveness and safety in the treatment of moderate-to-severe AD.

## 2. Materials and methods

### 2.1. Search strategy

This SR and meta-analysis was guided by Preferred Reporting Items for Systematic Reviews and Meta-Analyses (PRISMA) filing system.^[11]^ It was devoid of any publication bias as the protocol was registered in the PROSPERO database (Registration ID: CRD42024617230).^[12]^ There was a broad search on Google Scholar, Web of Science, Wiley Searching, Science Direct, PubMed, and EBSCO published after a specific date. To further reduce bias, searches were also conducted in clinical trial registries to discover unpublished studies or pending completed trials that had not been published. As with all meta-analysis, gray literature, such as institutional reports and dissertations, was consulted, but other unpublished studies that were consulted did not meet the criteria for inclusion. The following search terms were used: (“Upadacitinib” OR “Janus Kinase inhibitors” OR “JAK inhibitors”) AND (“Atopic Dermatitis” OR “Dermatitis”) AND (“Efficacy” OR “Safety” OR “Outcomes” OR “Adverse Effects” OR “Patient Tolerance” OR “Long-term Effects” OR “Treatment”).

### 2.2. Eligibility criteria

The methods of selection for this review, like inclusion and exclusion criteria, were based on the PICOTS framework (Population, Intervention, Comparator, Outcomes, Timing, and Setting)^[13]^:

1. Population: Patients whose conditions have been diagnosed as AD.
2. Intervention: The use of UPA or other JAK inhibitors.
3. Comparator: Placebo or other methods of interventions.
4. Outcomes: Among the primary outcomes were factors measuring effectiveness (i.e., disease severity reduction) and safety measures. Secondary outcomes included effects on patient tolerance and long term treatment impacts. Under these criteria, PICOTS, studies were included that matched our parameters. The excluded studies consisted of non-randomized controlled trials such as editorials, cross-sectional studies, letters, commentaries, reviews, or cohort studies, including the duplication of previously published articles. Furthermore, participants without a clear case of AD were excluded.

### 2.3. Study selection

**Figure 1.**
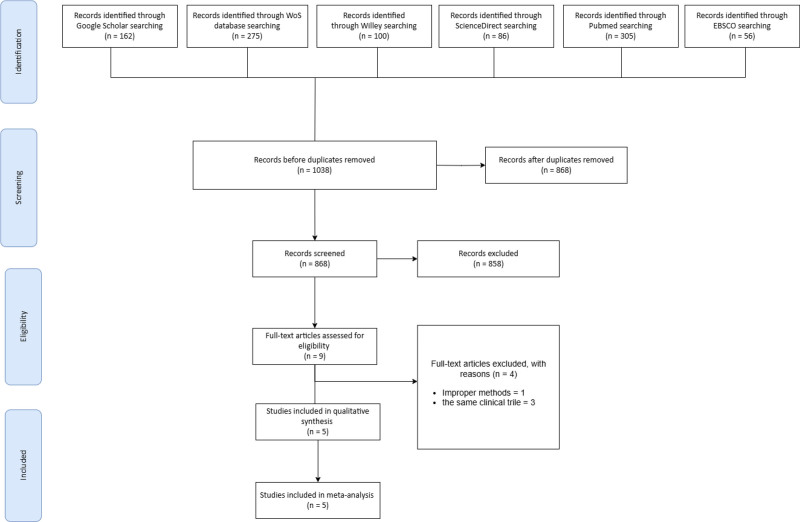
PRISMA flowchart. PRISMA = Preferred Reporting Items for Systematic Reviews and Meta-Analyses.

For precision and effectiveness, 2 separate reviewers employed Rayyan AI to screen every record title and abstract.^[14]^ The reviewers conducted full-text reviews for the selected papers and a third reviewer was consulted to resolve any disputes. The entire selection process is summarized in a PRISMA flow diagram.^[11]^ See Figure 1.

The studies included in the review met specific inclusion criteria, requiring them to be randomized controlled trials (RCTs) conducted on patients with a confirmed diagnosis of AD. They needed to evaluate the effectiveness of UPA or other JAK inhibitors compared to a placebo or another intervention and document at least one of the following outcomes: reduction in disease severity, safety profile, patient tolerance, or long-term treatment effects. Conversely, studies were excluded if they were non-RCTs, such as editorials, cross-sectional and cohort studies, letters, commentaries, reviews, or duplicate publications. Additionally, studies involving participants without a confirmed diagnosis of AD were not considered for inclusion.

### 2.4. Quality assessment

The methodological quality of included studies was assessed using the Revised Cochrane Risk of Bias Tool (RoB 2; Cochrane Collaboration, London, United Kingdom).^[15]^ Each study was evaluated for possible bias on several important aspects, such as random sequence generation, allocation concealment, blinding of subjects and personnel, blinding of outcome evaluators, and data including omission of outcome events, selective reporting, and others. Every study was rated as low, medium, or high risk of bias. Disagreements were settled by discussion or by using a third reviewer.

### 2.5. Data extraction

Two reviewers independently extracted data from the selected studies using a standardized data extraction form. The extracted data included:

1. The study information includes the author’s name, publication date, country of study, study type, sample size, and follow-up period, providing essential context for each study.
2. Participant details cover age range, gender distribution, disease severity, baseline parameters, and treatment duration, ensuring a clear understanding of the study population.
3. Intervention details specify the study and control groups, type of medication, dosage, duration, and method of administration, allowing for accurate comparisons.
4. Study outcomes which includes:
  a. The primary outcome evaluates the effectiveness of UPA or other JAK inhibitors for AD using validated scoring systems.
  b. Secondary outcomes assess safety profiles, adverse reactions, patient tolerance, long-term effects, and overall impact on quality of life.

### 2.6. Statistical analysis

Data analysis was performed using Review Manager (RevMan) version 5.4 (The Cochrane Collaboration, London, United Kingdom).^[16]^ A random-effects model was applied to calculate the standardized mean difference with 95% confidence intervals (CIs) for continuous outcomes. Heterogeneity among studies was assessed using the *χ*² test and *I*² statistic, with statistical significance set at *P* < .05.^[17]^

## 3. Results

A total of 1038 entries were identified from the systematic search conducted on 6 dissimilar databases, including Web of Science, Google Scholar, Wiley, Science Direct, PubMed, and EBSCO. After the removal of 170 duplicate records, 868 records were screened for inclusion criteria. This means that 858 articles found during screening based on the titles and abstracts excluded relevant outcomes or study designs in the process of assessing the 858 abstracts. Out of the 9 identified articles, 4 full-text articles were further excluded as they did not have a suitable methodological approach or reported results of the same clinical trial. Therefore, 4 and 5 articles were used for the meta-analysis, but in the qualitative synthesis, 5 articles were used. A PRISMA flow diagram of Figure 1 is presented to provide evidence about the identification and incorporation of studies involved in the SR and to explain why particular studies were excluded at different points.

### 3.1. Baseline characteristics of included studies

The included studies varied in design, sample size, and follow-up duration. Most were RCTs with follow-up periods of 12 to 16 weeks. Participants’ mean age ranged from 6 to 36 years, with baseline EASI scores between 28 and 32. Comparators included placebo and dupilumab, depending on the study. See Table 1.

**Table 1:** Baseline characteristics of the included studies.

Study ID	Authors (yr)	Country	Design	Sample size (intervention/control)	Follow-up duration	Age (mean ± SD)	Baseline disease severity (mean EASI/IGA)	Comparator
Study 1	Guttman-Yassky et al ^[22]^	USA, Germany, Canada	RCT	167 (42 per dose group, 41 placebo)	16 weeks	34 ± 5 yr	EASI ~32	Placebo
Study 2	Qian et al^[20]^	Global	Phase 1 open-label	35 (pediatric)	12 weeks	6 ± 2 yr	EASI ~28	None
Study 3	Katoh et al^[8]^	Japan	Phase 3 RCT	272 (120 UPA 15 mg, 122 UPA 30 mg, 90 placebo)	16 weeks	36 ± 5 yr	EASI ~31	Placebo
Study 4	Blauvelt et al^[18]^	Multinational (129 centers)	Phase 3b RCT	673 (342 UPA 30 mg, 331 dupilumab 300 mg)	16 weeks	36.2 ± 14.4 yr	EASI ~31	Dupilumab
Study 5	Silverberg et al^[19]^	Multinational	RCT	920 (458 UPA, 462 DUPI)	16 weeks	35 ± 5 yr	EASI ~30	Dupilumab

EASI = Eczema Area and Severity Index, IGA = Investigator’s Global Assessment, RCT = randomized controlled trial, SD = standard deviation, UPA = upadacitinib.

### 3.2. Risk of bias assessment

The risk of bias assessment indicated low selection bias across most studies. Performance bias was high in 3 studies, while detection bias remained low throughout. Attrition and reporting biases were generally low, except for one study with unclear attrition bias. See Table 2.

**Table 2:** Risk of bias assessment for the included studies.

Study ID	Selection bias	Performance bias	Detection bias	Attrition bias	Reporting bias
Guttman-Yassky et al^[22]^	Low (+)	High (−)	Low (+)	Unclear (?)	Low (+)
Qian et al^[20]^	Unclear (?)	High (−)	Low (+)	Low (+)	Low (+)
Katoh et al^[8]^	Low (+)	Low (+)	Low (+)	Low (+)	Low (+)
Silverberg et al^[19]^	Low (+)	High (−)	Low (+)	Low (+)	Low (+)
Blauvelt et al^[18]^	Low (+)	High (−)	Low (+)	Low (+)	Low (+)

### 3.3. Primary outcomes of the meta-analysis

#### 3.3.1. EASI-75 (75% improvement)

**Figure 2.**
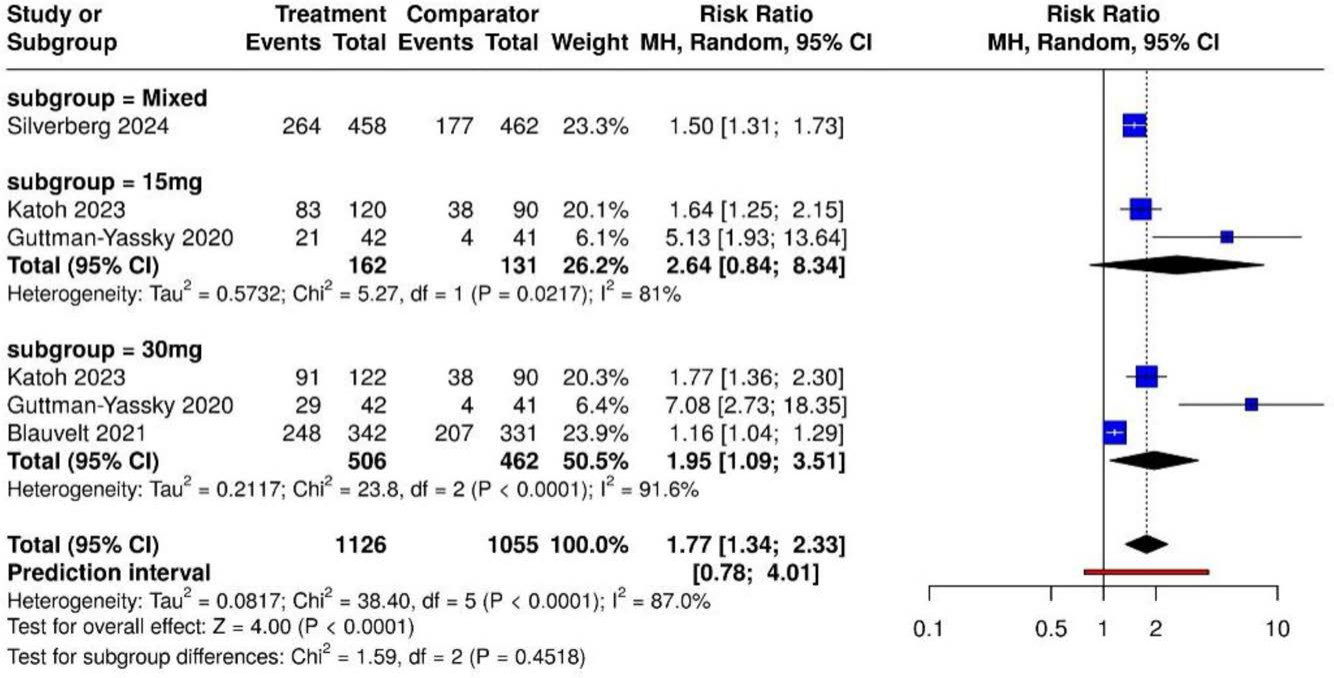
Forest plot showing the effect of upadacitinib 15 mg and 30 mg on EASI-75 response rates compared to placebo or dupilumab. EASI = Eczema Area and Severity Index.

The forest plot (Fig. 2) shows that UPA significantly increased the percentage of participants achieving at least a 75% reduction in EASI scores compared to placebo or dupilumab (total relative risk [RR] = 1.77, 95% CI: 1.34–2.33). Heterogeneity was substantial (*I*² = 87.0%). Subgroup analysis revealed that UPA 15 mg had a pooled RR of 2.64 (95% CI: 0.84–8.34), while the 30 mg subgroup demonstrated a pooled RR of 1.95 (95% CI: 1.09–3.51). Although both doses showed efficacy, statistical significance was more robust for the 30 mg group. The test for subgroup differences was not significant (*χ*² = 1.59, df = 2, *P* = .4518), indicating similar benefit across doses.

#### 3.3.2. EASI-90 (90% improvement)

**Figure 3.**
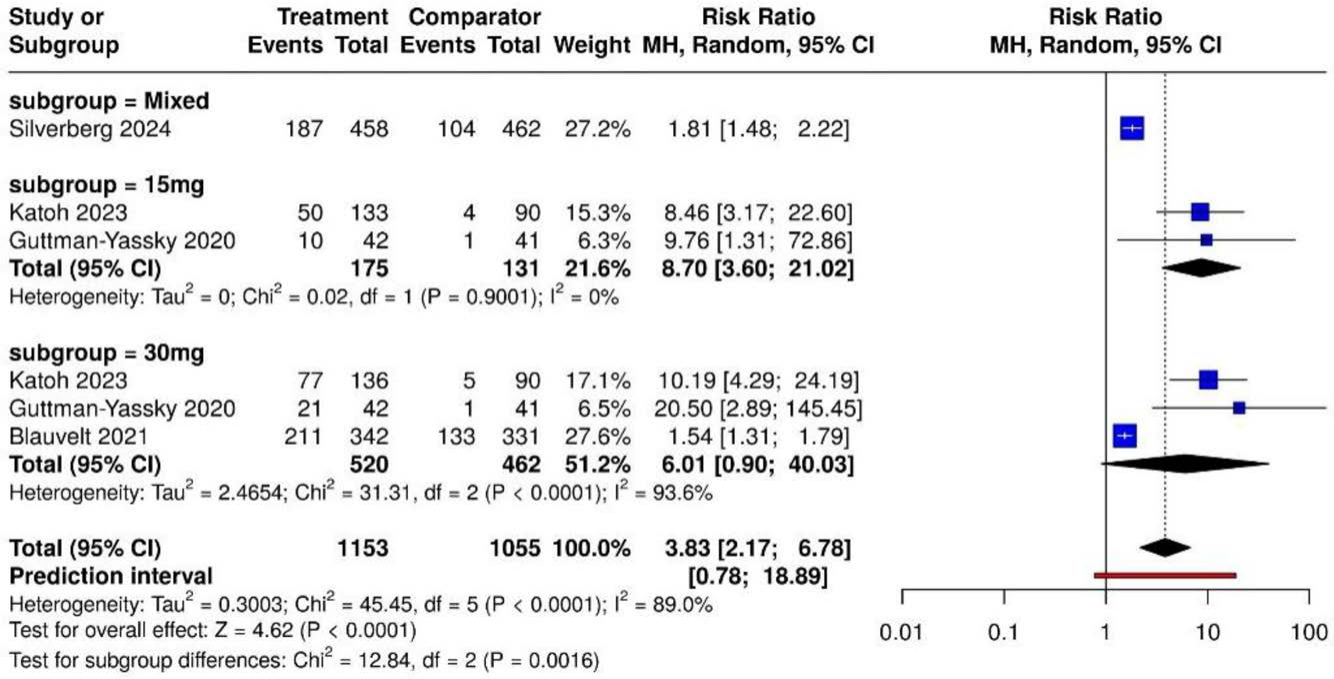
Forest plot showing the effect of upadacitinib 15 mg and 30 mg on EASI-90 response rates compared to placebo or dupilumab. EASI = Eczema Area and Severity Index.

Figure 3 demonstrates that UPA significantly improved EASI-90 outcomes compared to placebo or dupilumab (total RR = 3.83, 95% CI: 2.17–6.78). Heterogeneity was high (*I*² = 89.0%). Subgroup analysis showed that UPA 15 mg produced a pooled RR of 8.70 (95% CI: 3.60–21.02), while the 30 mg subgroup had a pooled RR of 6.01 (95% CI: 0.90–40.03). Although the 15 mg subgroup showed a numerically higher RR, confidence intervals were wider and overlapping. The test for subgroup differences was statistically significant (*χ*² = 12.84, df = 2, *P* = .0016), indicating that the dose level significantly influenced treatment effect.

#### 3.3.3. Pruritus numeric rating scale (NRS)

**Figure 4.**
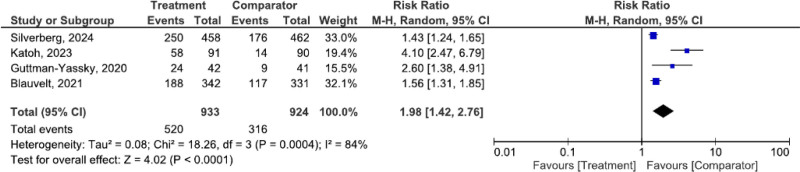
Pooled analysis of pruritus improvement, showing significant reduction in pruritus numeric rating scale (NRS) scores.

The pruritus NRS improvement (≥4-point reduction) is illustrated in Figure 4. The pooled effect size was 1.98 (95% CI: 1.42–2.76, *P* < .0001) with high heterogeneity (*I*² = 84%).

#### 3.3.4. Adverse events (AEs)

**Figure 5.**
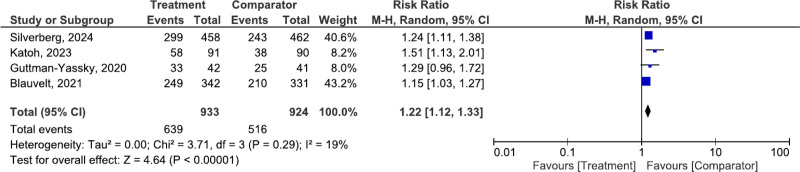
Risk ratio analysis of adverse events associated with upadacitinib, indicating its overall safety profile.

Figure 5 presents the analysis of AEs. The pooled risk ratio was 1.22 (95% CI: 1.12–1.33, *P* < .00001) with low heterogeneity (*I*² = 19%), indicating that most AEs were mild or moderate.

#### 3.3.5. Excluded Study: Qian et al. (2024)

*Qian et al. (2024*): The study design is an open label, pharmacokinetic study in pediatric patients rather than comparative efficacy outcomes. Thus, the Qian et al (2024) paper was thus excluded from the meta analysis. However, the study did yield some interesting data around which UPA proved to be especially beneficial in the pediatric population of these patients with severe AD, as shown by the improvement in EASI 75 and pruritus score E. Mean percentage decline was 62%. Additionally, many of the respondents showed *a* ≥ 4 point change in their pruritus NRS. These results support a possible effectiveness of UPA in children but further randomized controlled trials are needed in order to rely on these statements.

## 4. Discussion

This SR and meta-analysis assessed the efficacy and safety of UPA, a selective JAK inhibitor, in the treatment of moderate-to-severe AD. The findings revealed that UPA significantly improved disease severity, as indicated by higher EASI-75 (RR = 1.77, 95% CI: 1.34–2.33) and EASI-90 (RR = 3.83, 95% CI: 2.17–6.78) response rates compared to placebo or dupilumab. Notably, pruritus reduction was observed as early as Day 2, with significant improvements maintained through Week 16.^[8,18,19]^ Long-term data from the Rising Up study confirmed the durability of these effects, with efficacy sustained through Week 112 without loss of therapeutic benefit.^[8]^ AEs were mostly mild to moderate, with the most common being nasopharyngitis and acne. Serious AEs were rare (RR = 1.22, 95% CI: 1.12–1.33), reinforcing the favorable safety profile of UPA.^[20,21]^ These findings support its potential as an effective systemic treatment for moderate-to-severe AD, particularly in patients with inadequate responses to existing biologics or other therapies.

The results align with previous studies evaluating UPA’s efficacy. Blauvelt et al reported that UPA was more effective than dupilumab, achieving higher EASI-100 rates (28.4% vs 7.9% at Week 16), demonstrating superior potential for complete skin clearance.^[18]^ Similarly, Silverberg et al found that UPA provided more rapid and pronounced improvements in pruritus and EASI-90 scores compared to dupilumab, establishing its role as a competitive systemic therapy.^[19]^ The meta-analysis identified notable heterogeneity in EASI outcomes (*I*² = 87.0% for EASI-75 and 89.0% for EASI-90), likely attributable to differences in study design, baseline severity, and dosage. Subgroup analysis clarified that dosing plays a critical role in therapeutic effect. While both 15 mg and 30 mg doses of UPA were effective, the 30 mg dose consistently showed stronger and more statistically robust results for EASI-75 (RR = 1.95) and EASI-90 (RR = 6.01). However, the 15 mg dose showed a remarkably high RR for EASI-90 (8.70), possibly due to specific study contexts and smaller sample sizes. These findings support dose-dependent efficacy of UPA in AD, with a potential advantage for higher dosing in select patients. Blauvelt et al. also highlighted UPA’s effectiveness in difficult-to-treat cases, particularly in patients switching from dupilumab.^[21]^ Pediatric data from Qian et al further demonstrated EASI-75 achievement rates of 70% at 12 weeks, with a safety profile comparable to that in adult populations.^[20]^ Mechanistically, UPA’s selective JAK1 inhibition suppresses multiple cytokines implicated in AD pathogenesis, including IL-4, IL-13, and IL-31. This broad mechanism of action likely contributes to its rapid pruritus reduction compared to dupilumab, which specifically targets IL-4 and IL-13.^[22,23]^ Other JAK inhibitors, such as abrocitinib, exhibit similar efficacy but are associated with a higher incidence of gastrointestinal side effects, reinforcing UPA’s favorable efficacy-to-safety ratio.^[24]^

The robust efficacy of UPA appears to stem from its targeted JAK1 inhibition, which blocks key inflammatory pathways involving IL-4, IL-13, and IL-31. This mechanism may explain its rapid pruritus relief within the first 2 days and substantial skin clearance by Week 16.^[22]^ The pooled meta-analysis results further confirm this effectiveness, with an RR of 2.68 for EASI-90 responders. While AEs such as nasopharyngitis and acne were common, they were generally mild. Although rare, serious AEs necessitate careful monitoring, particularly in patients with predisposing risk factors.^[20]^ Long-term safety assessments, such as those in the Rising Up study, have not identified any new safety concerns over 112 weeks of treatment.^[8]^ Despite the promising results, the high heterogeneity observed across studies warrants cautious interpretation, as variations in baseline disease severity, study methodologies, and dosing regimens (15 mg vs 30 mg) may have influenced outcomes. To establish UPA’s definitive role in systemic AD treatment, further standardized trials and direct comparisons with other JAK inhibitors, such as abrocitinib, are necessary.^[24]^

The findings underscore the clinical significance of UPA as a treatment for moderate-to-severe AD. The high rates of EASI-75 and EASI-90 achievement indicate not only an overall reduction in disease severity but also a substantial proportion of patients attaining near-complete skin clearance. Additionally, its rapid and sustained pruritus relief highlights its potential to enhance patients’ quality of life significantly. UPA thus represents a valuable option for individuals who do not achieve adequate control with topical therapies or conventional systemic treatments. Its targeted action as a JAK inhibitor offers a more precise and potentially more effective alternative to existing therapies, making it particularly beneficial for patients seeking rapid symptom relief. Moreover, its relatively mild AE profile further supports its clinical utility. Studies evaluating real-world effectiveness reinforce these findings, demonstrating consistent efficacy and patient satisfaction. However, long-term safety monitoring remains essential, given the chronic nature of AD management.^[25,26]^

While the results are encouraging, several limitations must be considered. The high heterogeneity in outcomes, particularly pruritus and EASI improvements, suggests that differences in study populations, treatment protocols, and methodologies could affect the generalizability of findings. Additionally, some studies exhibited a risk of bias, particularly in performance measures, potentially leading to overestimation of treatment effects. Variations in patient and clinician expectations may have influenced reported outcomes, limiting their applicability to real-world settings.

Another key limitation is the relatively short follow-up duration in most studies, typically capped at 16 weeks. Since AD is a chronic condition requiring long-term management, extended studies are necessary to fully assess UPA’s durability, potential cumulative adverse effects, and the need for dose adjustments over time. One study emphasized the importance of long-term safety evaluations to establish the sustained benefits and risks of UPA.^[27]^

Future research should address these limitations and expand the evidence base for UPA. Key recommendations include conducting long-term studies to evaluate sustained efficacy and safety, as longer follow-ups are crucial for chronic disease management.^[20,25]^ Standardizing outcome measures, particularly EASI and pruritus assessments, would reduce variability across studies and enhance comparability. Expanding research to include a broader demographic range, such as pediatric and elderly populations, would improve understanding of how different patient groups respond to treatment.^[25]^ Collecting real-world data on UPA’s effectiveness in routine clinical settings would further bridge the gap between trial results and everyday patient experiences. Finally, head-to-head trials directly comparing UPA with standard treatments like dupilumab and other JAK inhibitors are needed to refine clinical decision-making.^[18,21,24]^

## 5. Conclusion

This SR and meta-analysis highlight the strong efficacy and favorable safety profile of UPA in treating moderate-to-severe AD. The findings demonstrate significant improvements in disease severity, with higher EASI-75 (RR = 1.77, 95% CI: 1.34–2.33) and EASI-90 (RR = 3.83, 95% CI: 2.17–6.78) response rates compared to placebo or dupilumab. Rapid symptom relief was observed as early as Day 2, with long-term efficacy sustained up to 112 weeks. While mild to moderate AEs were common, serious adverse reactions were infrequent, supporting its favorable risk-benefit profile. The JAK1-selective inhibition mechanism likely contributes to its rapid and sustained effects, making it a promising systemic therapy for patients who do not respond adequately to existing treatments. However, challenges such as study heterogeneity (*I*² = 87.0% for EASI-75, 89.0% for EASI-90), limited follow-up durations, and potential bias necessitate further research. Long-term trials, standardized methodologies, and real-world data will be essential in validating these findings and optimizing UPA’s role in clinical practice.

## Acknowledgments

We acknowledge the contributions of all researchers whose studies were included in this review and appreciate the support received during the preparation of this manuscript.

## Author contributions

**Conceptualization:** Amr Molla, Mohammed Alahmadi, Sara Alghamdi, Abdulsalam Humedi, Maryam Alfaraj, Basel Bakhamees, Samia Khalil, Salma Alhussaini.

**Data curation:** Amr Molla, Mohammed Alahmadi, Sara Alghamdi, Abdulsalam Humedi, Maryam Alfaraj, Basel Bakhamees, Samia Khalil, Salma Alhussaini.

**Formal analysis:** Amr Molla, Mohammed Alahmadi, Sara Alghamdi, Abdulsalam Humedi, Maryam Alfaraj, Basel Bakhamees, Samia Khalil, Salma Alhussaini.

**Investigation:** Amr Molla, Mohammed Alahmadi, Sara Alghamdi, Abdulsalam Humedi, Maryam Alfaraj, Basel Bakhamees, Samia Khalil, Salma Alhussaini.

**Methodology:** Amr Molla, Sara Alghamdi, Maryam Alfaraj, Basel Bakhamees, Samia Khalil, Salma Alhussaini.

**Project administration:** Amr Molla.

**Resources:** Amr Molla, Maryam Alfaraj, Basel Bakhamees, Samia Khalil.

**Software:** Amr Molla, Mohammed Alahmadi, Sara Alghamdi, Abdulsalam Humedi, Maryam Alfaraj, Basel Bakhamees, Samia Khalil, Salma Alhussaini.

**Supervision:** Amr Molla, Salma Alhussaini.

**Validation:** Amr Molla, Sara Alghamdi, Abdulsalam Humedi, Maryam Alfaraj, Basel Bakhamees.

**Visualization:** Amr Molla, Mohammed Alahmadi, Sara Alghamdi, Abdulsalam Humedi, Maryam Alfaraj, Basel Bakhamees, Samia Khalil, Salma Alhussaini.

**Writing – original draft:** Amr Molla, Mohammed Alahmadi, Sara Alghamdi, Abdulsalam Humedi, Maryam Alfaraj, Basel Bakhamees, Samia Khalil, Salma Alhussaini.

**Writing – review & editing:** Amr Molla, Mohammed Alahmadi, Sara Alghamdi, Abdulsalam Humedi, Maryam Alfaraj, Basel Bakhamees, Samia Khalil, Salma Alhussaini.
